# Targeting the *Aspergillus flavus p2c* gene through host-induced gene silencing reduces *A. flavus* infection and aflatoxin contamination in transgenic maize

**DOI:** 10.3389/fpls.2023.1150086

**Published:** 2023-05-09

**Authors:** Yenjit Raruang, Olanike Omolehin, Dongfang Hu, Qijian Wei, Surassawadee Promyou, Lidiya J. Parekattil, Kanniah Rajasekaran, Jeffrey W. Cary, Kan Wang, Zhi-Yuan Chen

**Affiliations:** ^1^ Department of Plant Pathology and Crop Physiology, Louisiana State University Agricultural Center, Baton Rouge, LA, United States; ^2^ Food and Feed Safety Research Unit, United States Department of Agriculture – Agricultural Research Service, Southern Regional Research Center, New Orleans, LA, United States; ^3^ Faculty of Natural Resources and Agro-Industry, Kasetsart University, Sakonnakhon, Thailand; ^4^ Department of Agronomy, Iowa State University, Ames, IA, United States

**Keywords:** *Aspergillus flavus* 1, polygalacturonase (*p2c*) 2, host induced gene silencing 3, aflatoxin resistance 4, transgenic maize 5, small RNA 6, breeding 7

## Abstract

*Aspergillus flavus* is an opportunistic fungal pathogen that infects maize and produces aflatoxins. Using biocontrol or developing resistant cultivars to reduce aflatoxin contamination has only achieved limited success. Here, the *A. flavus* polygalacturonase gene (*p2c*) was targeted for suppression through host-induced gene silencing (HIGS) to reduce aflatoxin contamination in maize. An RNAi vector carrying a portion of the *p2c* gene was constructed and transformed into maize B104. Thirteen out of fifteen independent transformation events were confirmed to contain *p2c*. The T2 generation kernels containing the *p2c* transgene had less aflatoxin than those without the transgene in six out of eleven events we examined. Homozygous T3 transgenic kernels from four events produced significantly less aflatoxins (*P ≤* 0.02) than the kernels from the null or B104 controls under field inoculation conditions. The F1 kernels from the crosses between six elite inbred lines with P2c5 and P2c13 also supported significantly less aflatoxins (*P ≤* 0.02) than those from the crosses with null plants. The reduction in aflatoxin ranged from 93.7% to 30.3%. Transgenic leaf (T0 and T3) and kernel tissues (T4) were also found to have significantly higher levels of *p2c* gene-specific small RNAs. Further, homozygous transgenic maize kernels had significantly less fungal growth (27~40 fold) than the null control kernels 10 days after fungal inoculation in the field. The calculated suppression of *p2c* gene expression based on RNAseq data was 57.6% and 83.0% in P2c5 and P2c13 events, respectively. These results indicate clearly that the reduced aflatoxin production in the transgenic kernels is due to RNAi-based suppression of *p2c* expression, which results in reduced fungal growth and toxin production.

## 1 Introduction

Maize (*Zea mays* L.) is one of the major crops susceptible to *Aspergillus flavus* infection and subsequent contamination with aflatoxins, the most potent carcinogenic secondary metabolites produced in nature (Castegnaro and McGregor, 1998). Aflatoxin is known to cause serious negative health impacts in both humans and domestic animals including liver cancer, cirrhosis, hepatitis, reproductive defects, and acute aflatoxicosis (Shephard, 2008). Due to the toxic and carcinogenic effects of aflatoxins, many countries have established strict regulations on the permissible levels of aflatoxin in food and feed, such as in the U.S., which the Food and Drug Administration (FDA) has set a limit not to exceed 20 µg/kg (ppb) for grains intended for human consumption (Park and Liang, 1993). The annual economic losses due to aflatoxin contamination in maize was estimated to be more than $500 million in the United States (Vardon et al., 2003).

Currently, complete elimination of aflatoxin contamination in maize is not feasible due to lack of effective management tools. Biocontrol is the only available tool that has shown effectiveness in reducing aflatoxin contamination in the field (Cotty and Bayman, 1993; Cotty et al., 2007; Jaime-Garcia and Cotty, 2007), but its efficacy depends a lot on the environmental conditions and the time of application (Moore et al., 2011). Although past efforts in screening various germplasm collections have identified sources of resistance to aflatoxin contamination in Mp420, Mp313E, Mp715, Mp717 and GT-MAS:gk (King and Scott, 1982; Widstrom et al., 1987; Scott and Zummo, 1988; Scott and Zummo, 1992; McMillian et al., 1993; Williams and Windham, 2001; Williams and Windham, 2006), no major progress has been made in transferring the polygenic resistance trait into elite breeding lines (Warburton et al., 2011; Mylroie et al., 2013), which remain susceptible to aflatoxin contamination.

RNA interference (RNAi) is a natural biological process in which the small interfering RNA (siRNA) molecules produced from double stranded (ds) RNAs by DICER enzyme bind to its target gene in a sequence specific manner and interfere with its transcription or translation (Hamilton et al., 2002; Dykxhoorn et al., 2003). Plants use this process to regulate growth and development and defend against pathogens. Several studies found that host produced siRNAs not only can suppress expression of endogenous genes, but also can be taken up by the invading pathogen and interfere with its growth and disease development, thus providing a novel approach for plant disease management (Großhans and Filipowicz, 2008).

The major breakthrough in reducing plant fungal diseases using RNAi came from the studies by Nowara et al. (2010) and Tinoco et al. (2010). Tinoco et al. (2010) suppressed the GUS expression in the *Fusarium verticillioides* using dsRNA targeting the GUS gene produced from the transgenic tobacco plants. Nowara et al. (2010) transgenically expressed a silencing construct to target the fungal effector *Avra10* in susceptible barley and wheat and successfully reduced the infection of powdery mildew fungus *Blumeria graminis*. This suppression of pathogen gene expression using siRNAs produced in the host is called host induced gene silencing (HIGS), which has been successfully shown to reduce fungal infection in various crops. For example, targeting fungal cytochrome P450 lanosterol C-14α-demethylase involved in ergosterol production in *Fusarium graminearum* through HIGS reduced head blight in barley (Koch et al., 2013). Suppression of a crucial *Fusarium graminearum* chitin synthase gene, (*Chs*) 3b, conferred durable resistance in wheat to Fusarium head blight and seedling blight with up to 85% reduction in deoxynivalenol (DON) toxin levels (Cheng et al., 2015). A similar study by Wang et al. (2020) reported that silencing of genes encoding a critical regulator of DON biosynthesis, a key transcription factor and an essential phosphatase in *Fusarium graminearum* resulted in reduced Fusarium head blight and DON production in wheat. Panwar et al. (2018) reported suppressing the wheat leaf rust fungus *Puccinia triticina* when genes involved in pathogenicity were targeted through transgenically expressed dsRNA. Ghag et al. (2014) and Dou et al. (2020) showed that transgenic banana producing siRNAs targeting vital fungal genes increased its resistance against *Fusarium oxysporum* f.sp. *cubense*. Jahan et al. (2015) demonstrated successful control of *Phytophthora infestans* in potato using the same strategy. Effective management of rice blast disease was also achieved by silencing *Magnaporthe oryzae* pathogenesis genes encoding membrane-bound adenylate cyclase and mitogen-activated protein kinase that play important roles in infectious hyphal growth and appressorium formation (Zhu et al., 2017). Song and Thomma (2018) reported suppression of Verticillium wilt in tomato and *Arabidopsis.* These studies demonstrated that small RNAs produced in host plants to target specific genes of a pathogen can be effectively used to manage plant diseases. In addition, this approach has been successfully used to reduce aflatoxin production in maize by targeting *aflR*, *aflC*, *aflM, alk*, or *amy1* of *A. flavus* (Masanga et al., 2015; Thakare et al., 2017; Gilbert et al., 2018; Raruang et al., 2020; Omolehin et al., 2021).

Here, we report the development of transgenic maize lines that can reduce *A. flavus* infection and aflatoxin production by employing this HIGS strategy. The *A. flavus p2c* gene, which encodes the polygalacturonase (also known as pectin depolymerase or pectinase) that is involved in breaking down pectin in plant cell walls during fungal colonization, was selected as the target gene for silencing. This pectinase plays an important role in the infection of cotton balls by *A. flavus* (Cleveland and McCormick, 1987) and is associated with the aggressiveness of different *A. flavus* isolates (Cleveland and Cotty, 1991; Shieh et al., 1997; Mellon and Cotty, 2004). Its importance in other pathogens in causing soft rot disease has been well documented (Collmer and Keen, 1986).

The aim of this study was to reduce *A. flavus* colonization during infection of maize by suppressing the highly expressed *p2c* gene through HIGS to subsequently reduce aflatoxin contamination. A HIGS vector was first constructed and transformed into maize B104, and the resulting transgenic kernels of heterozygous and homozygous plants were evaluated under laboratory and field inoculation conditions for five generations. Reduced aflatoxin contamination was observed in both homozygous transgenic B104 kernels as well as in kernels of F1 crosses of these lines with elite inbred lines. This increase in aflatoxin resistance appears to correlate with the presence of high levels of *p2c* gene specific small RNA being produced in transgenic maize tissues as well as the significantly reduced fungal growth in the inoculated homozygous transgenic kernels compared to the null controls.

## 2 Materials and methods

### 2.1 Construction of HIGS construct for suppressing *p2c* gene expression

*P2c* gene from *A. flavus* AF13 (accession number U05015) was selected as a potential target for suppression using a Gateway based vector previously constructed by Chen at al. (2010a). The 5′ and 3′ arms were amplified using PCR with homologous recombination sites attached to the end of the gene-specific primers (**Supplementary Table S1**), ligated into MultiSite Gateway pDONR vectors through BP clonase reactions, which were then recombined with pBS-d35S-attR4-attR3 and pDNR221-PR 10-intron-CmR (Chen et al., 2010a) to produce pBS-P2c-RNAi (pBS-d35S-attB4-5′ arm-attB1-PR 10 intronCmR-attB2-3′ arm-attB3) (**Supplementary Figure S1**) through LR clonase reaction following the same procedure described in our previous study (Raruang et al., 2020). The resulting vector was verified through sequencing before being digested with *Eco*RI and *Sac*I and ligated into the digested pTF102 vector to produce the final pTF102-P2c-RNAi construct, which was also verified through digestion before being used in maize transformation.

### 2.2 Transformation of dsRNA vector into maize and initial analysis of transgenic maize leaf tissues

The pTF102-P2c-RNAi vector DNA was transformed into *Agrobacterium tumefaciens* strain EHA101 at the Plant Transformation Facility (PTF), Iowa State University, which was then used to transform B104 immature zygotic embryos (Paz et al., 2006) according to the protocol described by Frame et al. (2000). The bialaphos was added in the media for transgenic selection. Pollen from B104 were used to pollinate all fifteen independent transformation events in Mar of 2013.

### 2.3 Verification of transformation and target gene expression

Ground leaf tissues (0.1 g) collected from all fifteen independent transformation events were extracted using a modified CTAB method to isolate genomic DNA according to Doyle and Doyle (1987), which was quantified using a NanoDrop (Thermo Scientific, Wilmington, DE, USA). One × PCR reaction was prepared as described previously (Raruang et al., 2020) using diluted genomic DNA as a template and P2c-F and P2c-R (**Supplementary Table S1**) as gene specific primers. For quantifying target gene expression, total RNA was isolated from the ground leaf tissue of all 15 events and the B104 wild type (negative control) and quantitative real-time PCR was performed using primer pair RT-P2c-F and RT-P2c-R (**Supplementary Table S1**) as described (Raruang et al., 2020). The target gene expression was normalized to that of 18S rRNA. The amplification efficiency for all primer pairs used in this study was also determined. Only positive transformation events were used in the following studies.

### 2.4 Evaluating aflatoxin accumulation in T1, T2, T3, and T4 generations of transgenic maize kernels and in F1 crosses with six elite inbred lines

Three events with high levels of gene expression (P2c8, P2c17 and P2c18) and four events with low gene expression (P2c5, P2c7, P2c13, and P2c25) were selected for the initial screening out of thirteen events of T1 generation transgenic maize kernels received from Iowa. Ten to fifteen kernels from each event were surface-sterilized, and inoculated with 7-day old conidia collected from *A. flavus* toxigenic strain AF13 (ATCC 96044, SRRC 1273) under the Kernel Screening Assay (KSA) conditions described by Brown et al. (1993). The aflatoxins were extracted and quantified using high performance liquid chromatography (HPLC) according to Sobolev and Dorner (2002) and Joshua (1993), respectively. After aflatoxin extraction, the ground powder from each kernel was used for genomic DNA isolated as described above to determine whether it contains the transgene or not.

Another fifteen T1 kernels from each of the above eleven events were planted in greenhouse, verified for the presence of transgene before being transplanted into the field at Louisiana State University Agricultural Center Botanic Gardens, Baton Rouge in the spring of 2015, for self-pollination to produce T2 seeds. Twenty-five kernels per event from the resulting T2 ears were tested for aflatoxin accumulation using KSA and for the presence of transgene as described above. Forty-five kernels/event of T2 seeds were planted in spring of 2016 for producing T3 seeds. Two of the events (P2c5 and P2c13) were increased to T4 in the field (60 kernel/event) in 2017. In 2018, homozygous and null lines of P2c5 and P2c13 events were crossed with six elite inbred line (3 non-stiff stalk: LH210, PHN46 and PHW79; and 3 stiff stalk: LH195, LH197 and PHG39) (Mikel, 2006) to determine whether the transgene can reduce aflatoxin production in the resulting crosses. Also in 2018, two more T2 events (P2c7 and P2c17) were increased, and aflatoxin levels were determined.

For evaluating aflatoxin resistance, 6 to 14 ears from each of the above lines were inoculated using a tree gun with a custom-made 15-gauge hypodermic needle with a side opening (Forestry Suppliers, Jackson, MS, USA) with 3.4 mL/per ear of *A. flavus* AF13 conidial suspension (4 × 10^6^ conidia/mL in 0.01% (w/v) SDS) two weeks after self-pollination at four injection sites in the mid-ear in 2016. The inoculum concentration for field inoculations was reduced to 1 × 10^5^ conidia/mL in 2017 and 2018 due to extremely high levels of aflatoxins detected in inoculated kernels from 2016. For 2016 and 2017, four mature kernels surrounding each needle injection site were collected and used as one sample for aflatoxin extraction and analysis. For the ears from the P2c7 and P2c17 events that were produced in 2018, kernels from the bottom half of the ears were harvested and ground as one sample for aflatoxin extraction and analysis to minimize aflatoxin variation from sample to sample. For crosses made in 2018, at least eight ears were collected per treatment. Kernels from the bottom half of each ear were collected and ground, and three subsamples were analyzed for aflatoxin as described below.

### 2.5 Aflatoxin extraction and quantification using HPLC

Aflatoxin was extracted from ground maize kernels (~60 mg-1000 mg) in a 50-mL flask containing 25 mL of an 80:20 methanol: water (HPLC grade) mixture according to Raruang et al. (2020). The extract was diluted 1:10 with 100% methanol in a 1.5 mL tube and filtered through an alumina-basic column (Sobolev and Dorner, 2002), collected in an autosampler vial, and quantified for aflatoxin levels using a Waters HPLC e2695 Separations Model containing a reverse-phase Nova-Pak C18 4 µm 3.9x150 mm column at 38°C linked to a 2475 FLR Detector (Waters Corp., Milford, MA, USA) as described in Sweany et al. (2011) with modifications (Raruang et al., 2020). The solvent for HPLC was 37.5% (v) Methanol: 62.5% (v) water at a 0.8 mL/min flow rate. Each sample run was 16 minutes with the aflatoxin B_1_ peak emerging at approximately 13.5 minutes. The aflatoxin quantification was performed using Empower 3 software (Waters Corp., Milford, MA, USA) with standard curves generated from serial diluted aflatoxin B_1_ standards (Sigma Aldrich, St. Louis, MO, USA) at 1, 5, 50, 500 and 1000 ng/mL.

### 2.6 Assessment of transgene copy number using real time PCR and droplet digital PCR

A zygosity test was conducted for all the events that confirmed positive for transformation. The heterozygous and homozygous plants among the T2, T3 and T4 seedlings were distinguished based on the relative ratio of *p2c* to the endogenous single copy alcohol dehydrogenase gene (*adh1*) determined through real-time PCR with gene specific primers and probes (**Supplementary Table S1**) according to the procedure described by Raruang et al. (2020). Three technical replicates were included for each sample. The initial *p2c/adh*1 ratio in the leaf tissue of T0 transgenic plants was used to determine the transgene copy number. Zygosity was calculated based on the threshold cycle (*Ct*) values of the target gene and *adh1* normalizer. If Δ*C_t_* (*C_t_* (target)- *C_t_* (endogenous)) of a T3 plant was less than that of the T0 plant, the T3 plant was considered homozygous (Bubner and Baldwin, 2004).

Droplet digital PCR to accurately quantify the transgene copy number was also performed on the genomic DNA extracted from the leaf tissue of the following samples: P2c5 (T0), P2c5 (T4), P2c7(T0), P2c7(T4), P2c13(T0), P2c13(T4), P2c17(T0), and P2c17(T4) using the same primer and probe set with *bar* gene as the target and the *adh1* gene as a reference at the Interdisciplinary Center for Biotechnology Research, University of Florida (Gainesville, FL, USA).

### 2.7 Small RNA library construction, sequencing and bioinformatics for detecting gene specific small RNA

Total RNAs were isolated from T0 leaf tissues of P2c5, P2c13, and P2c4 (null) events collected in 2013, from T3 leaf tissues of P2c13 homo, P2c4 (null), and B104 collected in 2016, and from the immature maize kernels of the T4 homozygous and null plants of P2c5 and P2c13 as well as B104 collected 2 weeks after self-pollination in 2017. The total RNA was isolated from the leaf tissues and ground kernel tissues using RNeasy Plant Mini Kit (QIAGEN, Hilden, Germany), and TRIzol respectively, according to the manufacturers’ instructions. The total RNA was quantified using a NanoDrop before being used for small RNA library synthesis. The indexed sRNA libraries were prepared as previously described (Raruang et al., 2020). The indexed sRNA libraries prepared from T3 leaf tissues in 2016 were sequenced on the Illumina HiSeq 2500 platform at the Genomic Science Laboratory at NC State University (Raleigh, North Carolina, USA), while the libraries prepared from T0 leaf tissues and T4 kernels in 2017 were sequenced on Illumina HiSeq 4000 at the Genomic Sequencing Core at UC Davis (Davis, CA, USA). The data analysis was performed as described by Hu et al. (2020). sRNA mapping figure was produced using R (R Development Core Team, 2013).

### 2.8 qPCR quantification of *A. flavus* biomass in maize kernel and RNAseq for determining target gene suppression

T4 generation homozygous and null P2c5 and P2c13 plants were self-pollinated, and the ears were inoculated with *A. flavus* (1×10^5^ conidia/mL in 0.01% (w/v) SDS) at 2 weeks after pollination as described above. At 10 days after inoculation, kernel surrounding each needle injection site were collected as one sample. The collected kernels from each ear were pulverized, and genomic DNA was extracted and quantified as described above. DNAs were diluted to 50 ng/µL and used as a template in qPCR to quantify the level of *its1* gene using specific primers (RT-Af2-F and RT-Af2-R, **Supplementary Table S1**), which is used as an indicator for fungal growth. The maize alcohol dehydrogenase gene (*adh1*) was used as an internal control to normalize the level of *A. flavus* biomass. The qPCR was performed as described (Raruang et al., 2020).

For determining target gene suppression, four kernels that surrounded the inoculation site were recovered 10 days after inoculation from T4 generation homozygous immature kernels of P2c5 and P2c13 ears with or without *A. flavus* inoculation and used for RNA isolation using RNeasy Plant Mini Kit (QIAGEN, Hilden, Germany). The RNA libraries were prepared with NEBNext Ultra Directional RNA Prep kit (Ipswich, MA, USA). The RNA libraries were sequenced on the HiSeq 2500 sequencer at the Genomic Science Laboratory at North Carolina State University (Raleigh, North Carolina, USA). The data analysis was performed as described above.

### 2.9 Statistical analysis

Statistical analysis was conducted using Excel (Microsoft Corp., Seattle, WA, USA) and SAS version 9.4 (Statistical Analysis System, SAS Institute, Cary, NC, USA). *Post-hoc* comparison of means was calculated using Tukey’s LSD means (Saxton, 1998). Significance in this study was defined by a confidence interval ≥95% (α = 0.05). The aflatoxin data from KSA assay of T1 and T2 generation of kernels were log transformed to equalize variation between samples of the experiment before statistical analysis while the rest of the aflatoxin data were used directly in statistical analysis without transformation.

## 3 Results

### 3.1 Construction and transformation of HIGS vector into maize

The details on the construction of the HIGS vector was described in **Supplementary Figure S1**. After assembling the *p2c* 5′ and 3′ arm and the PR10 intron with chloramphenicol selection marker (CmR) into pBS-d35S-attR4-attR3 through LR recombination to produce pBS-d35S-attB4-5′ arm-attB1-PR 10 intronCmR-attB2-3′ arm-attB3, the resulting construct was digested with EcoRV and EcoRI/SacI (**Supplementary Figure S2A**). The obtained fragment sizes were in agreement with the expected sizes upon digestion with these enzymes, which are 262, 1156, and 3589 bp; and 573, 1563, and 2871 bp, respectively. The T-DNA region from the above vector was also confirmed through sequencing with d35S-F, RNAi-R and PR10-F primers (**Supplementary Table S1**) before it was excised with Bam HI and Sac I, and inserted into the corresponding sites of pTF102. The final construct was verified through digestions with MfeI, SacI/EcoRI, EcoRV and EcoRI/EcoRV (**Supplementary Figure S2B**). The estimated fragment sizes based on DNA markers were as expected for a correctly assembled vector when digested with these enzymes, which are 299, 2086, and 8786 bp (MfeI); 573, 1563, and 9035 bp (SacI/EcoRI); 262, 668, 1156, and 9085 bp (EcoRV); and 262, 479, 668, 677, and 8461 bp (EcoRI/EcoRV). This construct is capable of producing a 220-bp *p2c* dsRNA with a 101-bp single-strand loop in the middle once it is transcribed and processed in the host plant.

**Figure 1:**
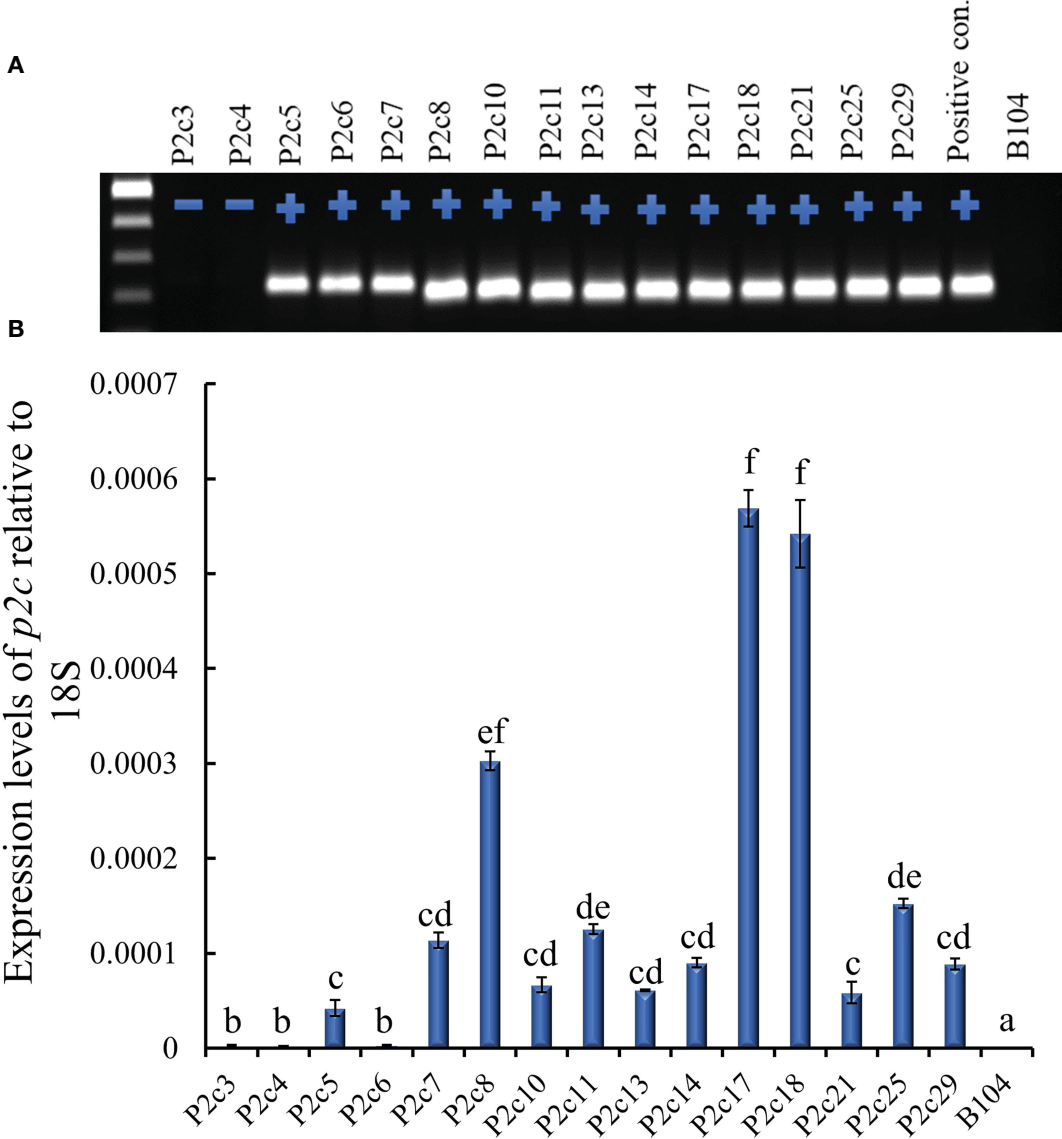
Determining the presence and level of expression of the target gene in the T0 *p2c* RNAi transgenic leaf tissues. **(A)**, PCR confirmation of the presence (+) or absence (-) of target gene in polygalacturonase (*p2c*) RNAi vector transformed T0 leaf tissues. P2c RNAi plasmid DNA was used as a positive control and the genomic DNA from maize line B104 was used as a negative (-) control. **(B)**, Expression of transgene *p2c* in the T0 leaf tissue of various transformation events relative to 18S rRNA using real time PCR. P2c3 and P2c4 are negative for the transgene. Events labeled with the same letters are not significantly different at *P ≤* 0.05.

The above construct was introduced into immature embryos of maize B104 line through *Agrobacterium* infection in September, 2012. Twenty-eight transgenic plants were produced from 15 independent transformation events and pollinated with pollen from B104 in March, 2013, and mature seeds were harvested from April to May, 2013. All events except P2c3 and P2c4 were confirmed for the presence of the *p2c* gene through PCR (**Figure 1A**). The highest *p2c* expression in T0 leaf tissues was detected in P2c8, P2c17, and P2c18 events and the lowest expressed was detected in P2c5, P2c6, and P2c21 (**Figure 1B**).

### 3.2 Characterization of T1 and T2 generations of transgenic seeds

**Figure 2:**
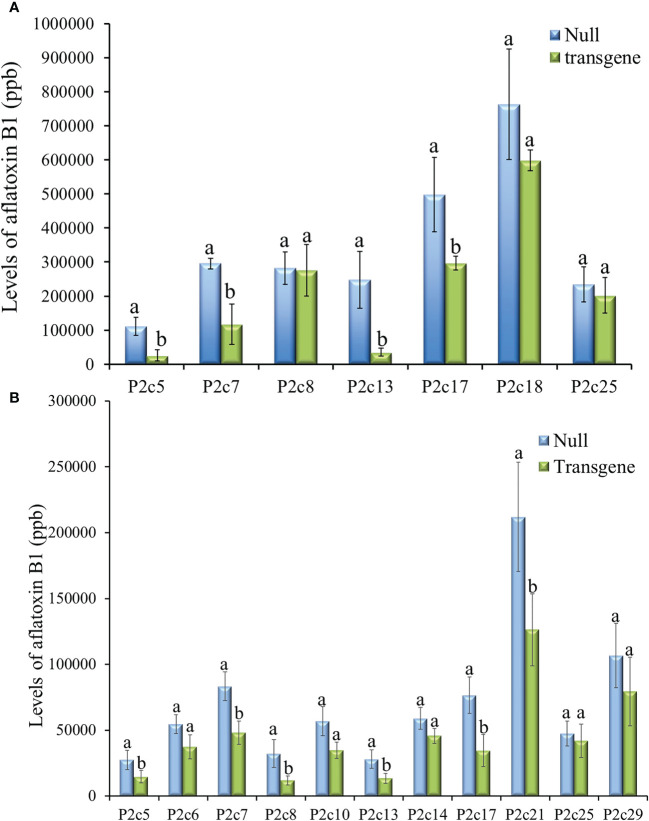
Aflatoxin production in the T1 **(A)** and T2 **(B)** generation of transgenic kernels containing *p2c* from different events compared to null seeds under kernel screening assay (KSA) conditions. Data presented here are the mean and standard errors of ten replicates for each event. Bars labeled with the same letters are not significantly different at *P ≤ 0.05.* Transgene represents the kernels that contain *p2c*. Null seeds for T1 are kernels from the same transformation events without the presence of *p2c*, and for T2 are segregating non-transgenic kernels from the same transformation events.

Twenty-three ears were produced from the 13 T0 events that were confirmed to contain *p2c*. The number of kernels per ear ranged from 12 to 246, and average kernel weight ranged from 0.17 to 0.26 g (**Supplementary Table S2**). T1 kernels from 7 events were examined for aflatoxin resistance through KSA. The transgenic kernels from four of the events (P2c5, P2c7, P2c13, and P2c17) were found to have significantly less aflatoxin than the kernels without the transgene (null) (**Figure 2A**). The reduction in aflatoxin ranged from 85.7% in P2c13 to 40.5% in P2c17. In an effort to conduct more comprehensive aflatoxin analyses, T1 seeds from the P2c5, P2c6, P2c7, P2c8 P2c10, P2c13, P2c14, P2c17, P2c21, P2c25, and P2c29 events were self-pollinated in the field in 2015 to produce T2 seeds. Significant aflatoxin reduction in the transgenic kernels of P2c5, P2c7, P2c8, P2c13, P2c17 and P2c21 events was observed when compared to their corresponding segregating non-transgenic null kernels (**Figure 2B**). The reduction in aflatoxin ranged from 63.3% for P2c8 to 40.4% for P2c21. The transgenic kernels from P2c5 and P2c13 were found to consistently perform better than P2c7 and P2c17 in both T1 and T2 generations. Due to the consistently higher aflatoxin resistance in kernels from P2c5 and P2c13 events, they were increased in the field of 2016 and 2017 to T3 and T4 generations, respectively.

### 3.3 Phenotypic evaluation of transgenic plants

**Figure 3:**
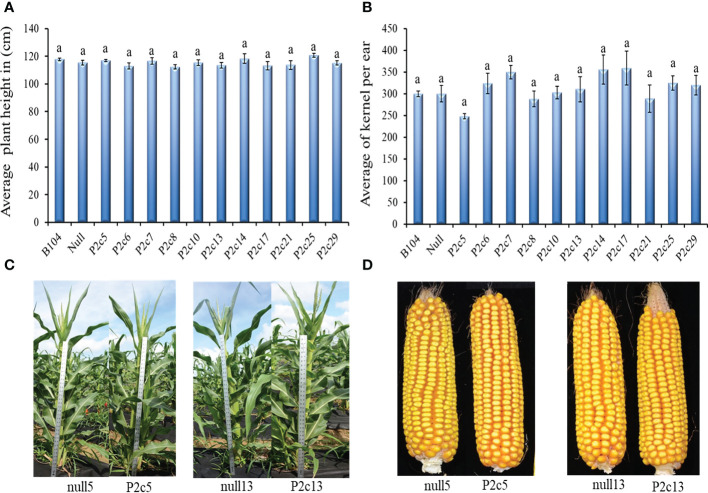
Phenotypic assessment of transgenic plants and mature cobs. **(A)** Average plant height in 11 events in comparison to the Null (P2c3 null) and B104 (wild type) controls 50 days after planting at T1 generation. **(B)** Average number of T2 generation kernels per cob in 11 events compared to Null (P2c3 null) and B104 (wild type). Vertical bars represent standard errors of the means. Means with the same letters were not significantly different at *P ≤ 0.*05. **(C)** Representative examples of plant height of null5 (P2c5 null), P2c5, null13 (P2c13 null), and P2c13 at 50 days after planting (T4). **(D)** Representative samples of dehusked mature maize cobs from of null5, P2c5, null13, and P2c13 at harvest (T4).

The potential impact of the presence of *p2c* gene and the process of *Agrobacterium* based transformation on maize plant growth and development was also evaluated. The height (T1 generation) of 8 to 10 plants at the silk stage and the number of kernels per ear (T2 generation) from 5-10 ears from 11 transgenic events, null, and B104 line were determined. Plant height and seed number per ear were not significantly different between transgenic and non-transgenic plants and also among different events (**Figures 3A, B**). The height at T3 generation and size of ears at T4 generation were not visually different either between transgenic plants and their null controls (**Figures 3C, D**).

### 3.4 Aflatoxin production in T3 and T4 generation homozygous seed

**Figure 4:**
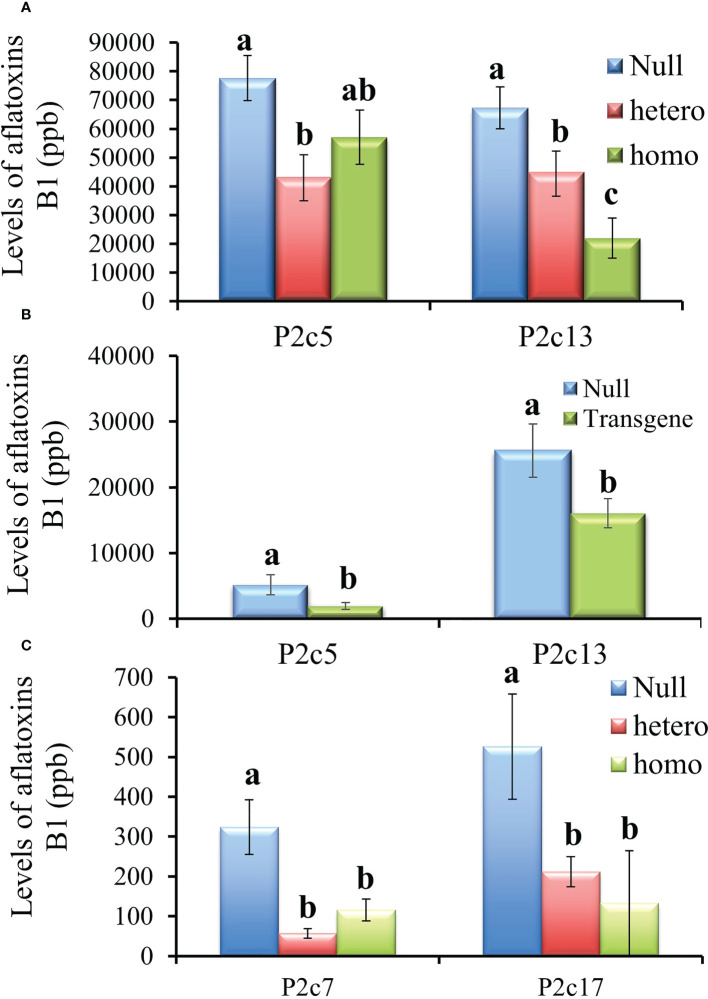
Aflatoxin production in the T3 generation kernels of four different events that were homozygous, heterozygous, and null for the transgene under field inoculation **(A, C)** and of two events (P2c5 and P2c13) under laboratory kernel screening assay conditions **(B)**. Null refers to the segregating non-transgenic kernels from the same event. Data are the mean and standard errors of 28-40 replicates for each event. Bars with the same letters are not statistically different at *P ≤* 0.05.

The T3 generation kernels heterozygous for *p2c* in P2c5 event in 2016 showed significantly lower levels of aflatoxin production than the kernels from the null (segregating non-transgenic) (*P*≤ 0.02) under field inoculation conditions. However, aflatoxin levels were not significantly different in the inoculated T3 kernels that were homozygous for *p2c* compared to that from the null control although it was lower (**Figure 4A**). Whereas for the event of P2c13, both the inoculated homozygous and heterozygous T3 generation kernels supported significantly lower levels of aflatoxin compared to kernels from the null (segregating non-transgenic) (*P ≤* 0.0002) (**Figure 4A**). The difference in aflatoxin resistance between the T3 homozygous and null kernels was also verified through laboratory KSA assays. Homozygous transgenic kernels from both P2c5 and P2c13 events produced significantly less aflatoxin compared to the null controls under KSA (**Figure 4B**). Overall, 40-80% reduction in aflatoxin production was observed for these two events under both the field and laboratory inoculations. The T3 generation homo and heterozygous kernels of P2c7 and P2c17 events produced and field-inoculated in 2018 also showed significantly lower levels of aflatoxin contamination compared to kernels from the null (segregating non-transgenic) with *P*≤ 0.0001 and *P ≤* 0.0026 respectively (**Figure 4C**), with an overall of 65-85% reduction in aflatoxin contamination.

**Figure 5:**
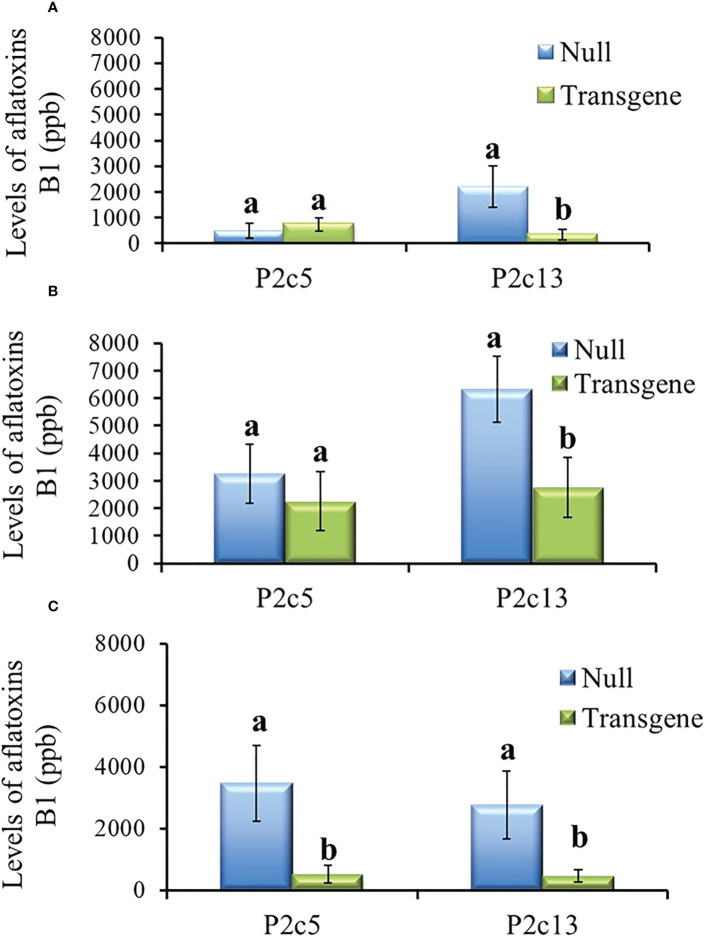
Aflatoxin production in the homozygous and null kernels of T4 generation from P2c5 and P2c13 events produced in first planting **(A, B)** and in second planting **(C)** under field inoculation **(A, C)** and KSA **(B)** conditions. Null represents the segregating non-transgenic kernels from the same event. Data presented here are the mean and standard errors of 28 replicates (for field inoculations) or 30 replicates (for KSA) from each event. Bars with the same letters are not significantly different at *P ≤* 0.05.

During the process of producing T4 generation homozygous transgenic kernels from P2c5 and P2c13 events in 2017, T3 kernels were planted twice. For the T4 generation kernels produced from the first planting, only the homozygous kernels from P2c13 showed significantly lower levels of aflatoxin than the kernels from the null (segregating non-transgenic) under both field inoculation (*P ≤* 0.0092) (**Figure 5A**) and KSA (*P ≤* 0.03) conditions (**Figure 5B**). For kernels produced from the second planting, homozygous kernels from both events (P2c5 and P2c13) produced significantly less aflatoxin than kernels from the null (*P ≤* 0.01 and *P ≤* 0.03, respectively) under field inoculation conditions (**Figure 5C**). A 70-90% reduction in aflatoxin production was observed for P2c13 event in both plantings and for P2c5 from the second planting under the field inoculation conditions (**Figures 5A, C**).

### 3.5 Zygosity and *p2c* transgene copy number estimation in different transformation events

In order to explain the consistently less efficacy in aflatoxin reduction observed from the P2c5 event compared to P2c13, their transgene copy number was determined. First, the number of seedlings with the presence (homozygous and heterozygous) or absence of the target gene (*p2c*) was determined in the segregating T3 seedlings developed from one ear of the self-pollinated P2c5, P2c7, P2c13, and P2c17 events using qPCR. The number of integrations of the target gene was then calculated based on chi-square analysis using a 95% confidence level (**Supplementary Table S3**). The results showed that P2c5 and P2c7 events contained more than one integration of the *p2c* target gene, whereas P2c13 and P2c17 events appeared to contain a single integration of *p2c* target gene.

To further verify this result, *p2c* copy number in the above transgenic lines was determined using droplet digital PCR. The calculated ratio of *bar*/*adh1* in genomic DNA extracted from P2c5(T0), P2c7(T0), P2c13(T0), and P2c17(T0) ranged from 0.52-0.95 (**Table 1**), confirming that the hemizygous plants from P2c13 and P2c17 events contain only one copy of the transgene, whereas the hemizygous plants from P2c5 and P2c7 events contain two-copies of the transgene. Plants from P2c5(T4), P2c7(T4), P2c13(T4), and P2c17(T4) events are confirmed homozygous for *p2c* based on the ratio of of *bar*/*adh1*, which ranged from 0.9 to 1.04 for P2c13 and P2c17 (single copy) and 1.83 to 1.85 for P2c5 and P2c7 (two copies) (**Table 1**). The droplet digital PCR data are consistent with our chi-square analysis (**Supplementary Table S3**).

**Table 1:** Gene copy number analysis through droplet digital PCR of genomic DNA from leaf tissues of T0 and T4 generation transgenic plants.

Events	*Bar* gene copy/20 µL	*Adh* gene copy/20 µL	*Bar/Adh*	Copy number
P2c5 (T0)	326	360	0.91	2 (hemi)
P2c7 (T0)	1112	1166	0.95	2 (hemi)
P2c13 (T0)	374	716	0.52	1 (hemi)
P2c17 (T0)	332	636	0.52	1 (hemi)
P2c5 (T4)	1160	628	1.85	2 (homo)
P2c7 (T4)	2306	1258	1.83	2 (homo)
P2c13 (T4)	798	880	0.90	1 (homo)
P2c17(T4)	618	590	1.04	1 (homo)

### 3.6 Detection of gene-specific small RNAs in transgenic leaf and kernel tissues

Small RNA sequencing was also performed to determine whether the reduced aflatoxin production in the homozygous transgenic kernels compared to the nulls was due to the presence of higher levels of *p2c* specific small RNA in the transgenic kernels. Small RNA libraries constructed from small RNAs isolated from T0 and T3 leaf tissues and from T4 kernel tissues were sequenced and analyzed. The total number of reads from each of the libraries varied greatly depending on the platform/facility where the sequencing was done, ranging from 0.4 million to 2.3 million for the libraries that were sequenced in 2017 at UC Davis, and ranging from 24.8 million to over 62 million for the libraries sequenced in 2016 at NC State (**Table 2**). However, after filtering out the reads that belong to maize genome, significantly high levels of *p2c*-specific small RNAs were observed in the T0 transgenic leaf tissues of P2c5 (5,032 reads) and P2c13 (1,119 reads) compared to the null (0 read). In the T3 leaf tissue, 857 *p2c*-specific reads were detected from P2c13 homo transgenic plants, compared to the 0 *p2c*-specific read observed for the null B104 or null P2c13 controls (**Table 2**). For the libraries prepared from the kernel tissue of P2c5 and P2c13 events, 179 and 467 *p2c*-specific reads were detected, respectively (**Table 2**), whereas only 4 and 30 *p2c*-specific reads were observed in libraries prepared from the null P2c5 and null P2c13 controls (**Table 2**), respectively. These data support that the reduced aflatoxin production in the homozygous transgenic kernels is due to the presence of high levels of *p2c*-specific small RNAs.

**Table 2:** Number of small RNA reads in leaf tissues and immature kernel tissues of transgenic and non-transgenic maize lines.

Tissue type*	Events	Total read	Reads aligned to maize genome	Reads aligned to *A. flavus >*1 times	Reads aligned to *A. flavus* 1 time	Reads aligned to *P2c*
Leaf tissue (T0) collected in 2013	P2c5	1,901,507	1,435,443	1,291	7,572	5,032
	P2c13	1,612,859	1,099,847	2,452	3,541	1,119
	P2c4 (null)	999,352	629,304	1,019	201	0
Leaf tissue (T3) collected in 2016	P2c13 homo	24,861,030	23,548,934	7,203	8,107	857
	P2c13 null	30,795,339	29,030,160	17,516	26,750	0
	B104 (WT)	62,902,688	61,179,007	5,285	6,236	0
Immature kernels (T4) collected in 2017	P2c5 homo	1,612,732	902,349	78	703	179
	P2c5 null	2,119,100	1,304,201	148	218	3
	B104 (WT)	1,367,547	796,105	670	732	0
	P2c13 homo	2,260,183	1,561,037	105	1,595	467
	P2c13 null	376,697	159,395	76	273	30
	B104 (WT)	1,336,547	769,105	670	732	0

* The small RNA libraries from T3 leaf tissues collected in 2016 were sequenced on Illumina HiSeq 2500 Platform at North Carolina State University and the small RNA libraries from T0 leaf tissues collected in 2013 and T4 immature kernel tissues collected in 2017 were sequenced on Illumina HiSeq 4000 Platform in 2017 at UC Davis.

**Figure 6:**
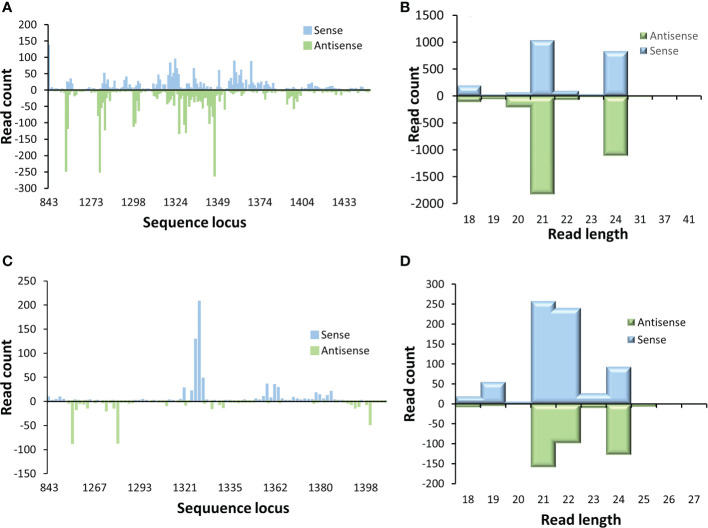
Small RNA profiling (RNAseq analysis) of *p2c* target genes in transgenic maize leaf tissues. Small RNA libraries constructed from total RNAs isolated from P2c5 **(A)** or P2c13 **(C)** leaf tissues containing the *p2c* gene were sequenced and mapped to the target sequence of *p2c*. Read length distribution of gene specific sRNAs from P2c5 **(B)** and P2c13 **(D)** mapped to *p2c*.

The locations in *p2c* gene where these small RNAs and the length distribution of these produced small RNAs were also examined. It appears that most of the small RNAs were generated from a few hot spots within the 220 bp target sequence region of the *p2c*. Also, the distribution pattern of these small RNAs along the *p2c* gene was similar between the P2c5 and P2c13 events (**Figures 6A, C**). However, the pattern of small RNA size distribution between these two events is different. The two most abundant small RNAs in P2c5 were 21 nt and 24 nt in length (**Figure 6B**), whereas in P2c13, the most abundant two small RNAs were 21 and 22 nt in length (**Figure 6D**). The ratio of 22 nt-long sRNAs specific to the *p2c* target region relative to other *p2c*-specific sRNAs in P2c13 is about 10-fold higher than that in P2c5 (0.30 *vs.* 0.03) (**Figures 6B, D**).

### 3.7 Analysis of fungal biomass and target *p2c* gene expression in *A. flavus* inoculated transgenic and control kernels

**Figure 7:**
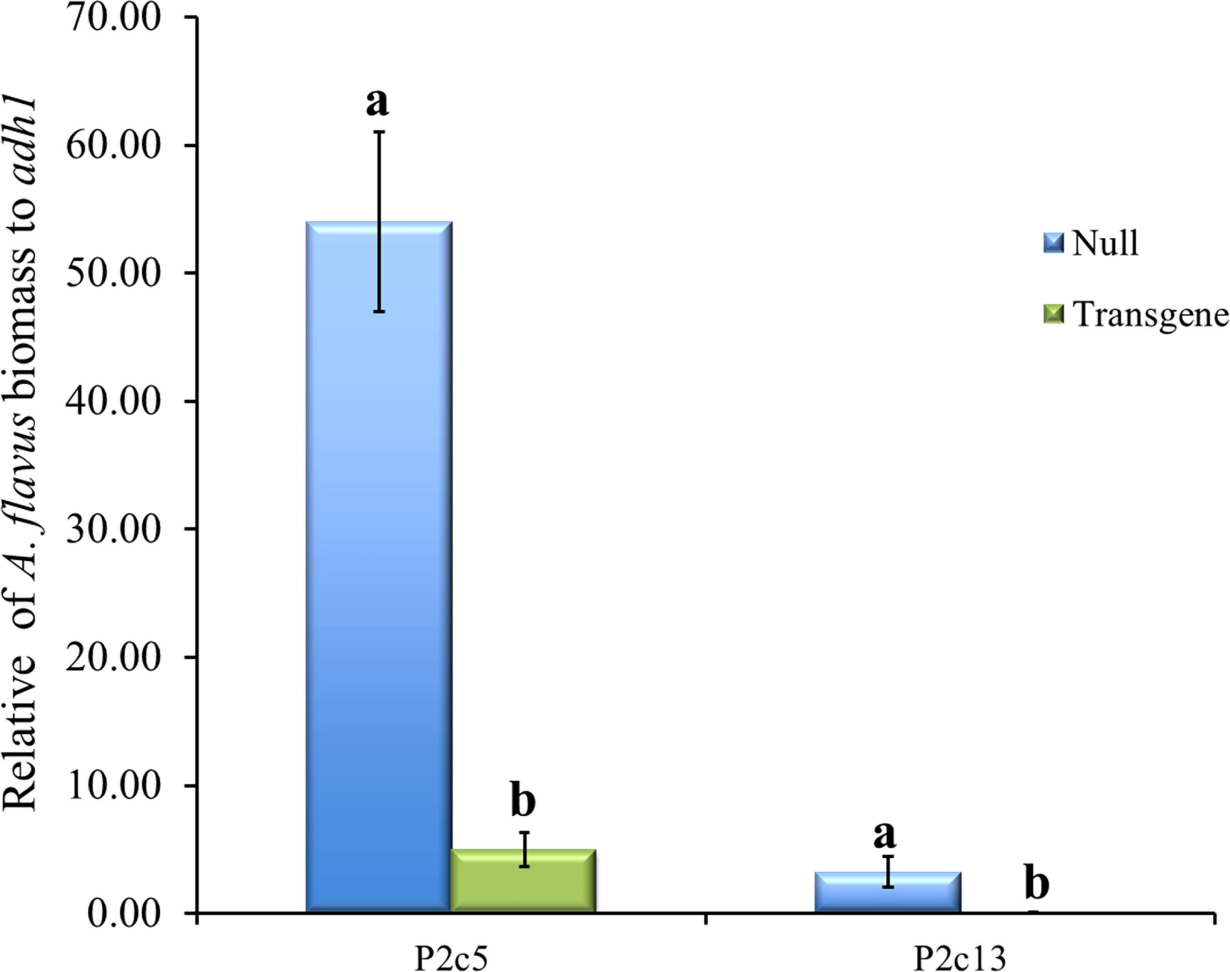
*A. flavus* growth in the homozygous transgenic and null kernels of T5 generation from two different events at 10 days after inoculation under field conditions. Null represents the segregating non-transgenic maize kernels from the same event. Fungal biomass was measured using real time PCR where the *A. flavus its1* gene was used for fungal quantification against the maize *adh1* gene. Data presented here are the mean and standard errors of 3 replicates from each event. Bars with the same letters are not significantly different at *P ≤* 0.05.

To determine whether the presence of high levels of gene specific *p2c* small RNA reduced *A. flavus* infection, *A. flavus* biomass was quantified using qPCR in samples of T5 generation transgenic and non-transgenic maize kernels 10 days after inoculation under field conditions. It was found that the kernels from transgenic events had significantly less *A. flavus* biomass than null (non-transgenic) in both events that were examined (**Figure 7**). Fungal growth in the inoculated homozygous transgenic maize kernels was reduced by 27 and 40-fold in P2c5 and P2c13 compared to that in the inoculated non-transgenic null 5 and null 13 kernels, respectively.

The total RNA isolated from the above kernel samples collected from inoculated and non-inoculated ears of P2c5 and P2c13 events were also sequenced to determine the *p2c* gene expression. The average number of *A. flavus* genes detected in the non-inoculated and inoculated kernel samples of P2c5 was 3489 and 7355 reads per million, respectively (**Supplementary Figure 3**). The *A. flavus* reads in the non-inoculated kernel samples are believed to come from natural infection since *A. flavus* is everywhere. At the meantime, the number of *p2c* specific reads detected in the non-inoculated and inoculated kernel samples was only 449 and 401 per million, respectively (**Supplementary Figure 3**). The ratio of total *A. flavus* reads to *p2c* reads is expected to remain similar if there is no suppression on the expression of *p2c*. The expected *p2c* specific reads in inoculated P2c5 would be 946.5 (=7355/3489 x 449). Therefore, the calculated percentage of target gene suppression is 57.6% ((1-401/946.5) x 100%) for the P2c5 event. The number of *A. flavus* genes detected in the non-inoculated and inoculated immature kernels of P2c13 event was 6105 and 6573 reads per million, respectively. Among them, only 230 and 42 reads per million from non-inoculated and inoculated immature kernels, respectively, were specifically aligned to *p2c* gene (**Supplementary Figure 3**). Following the same calculation, the percentage of target gene suppression in P2c13 event is 83.0% ((1- 42/247.6) x 100%). The significantly higher percentage of target gene suppression detected in P2c13 event than P2c5 is consistent with the higher aflatoxin resistance observed in P2c13 events compared to P2c5.

### 3.8 Aflatoxin resistance in elite inbred lines and F1 crosses with *p2c* silencing gene

**Figure 8:**
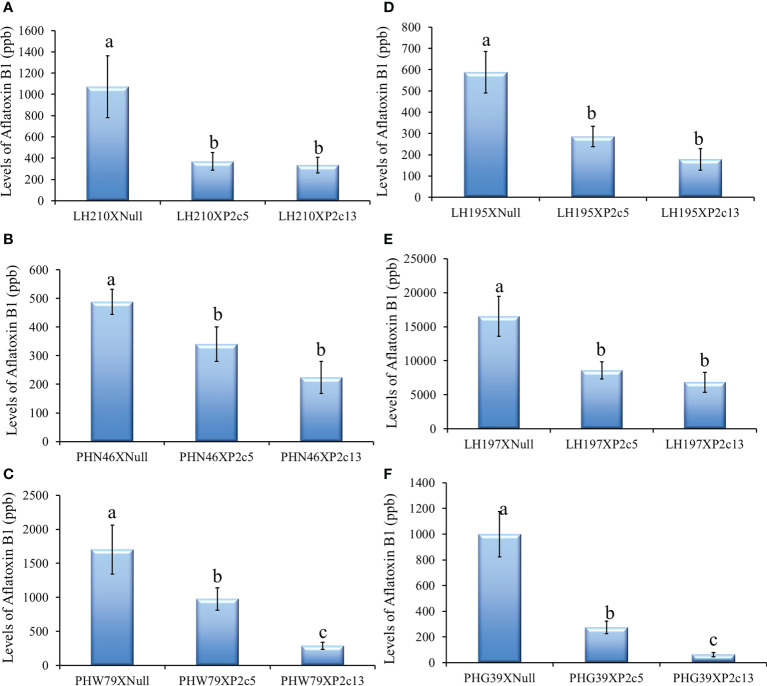
Aflatoxin production in the crosses between three non-stiff stalk LH210, PHN46 and PHW79 **(A–C)** and three stiff stalk LH195, LH197 and PHG39 **(D–F)** elite lines with P2c5 (homozygous, H, and null, N) or P2c13 (H and N) lines under field inoculation conditions. At least 8 ears per treatment were collected, and kernels from each half ear were ground and three subsamples were analyzed for aflatoxin levels using HPLC. The aflatoxin data for crosses between the elite lines and the P2c5 null and P2c13 null controls were combined for statistical analysis and presented as one bar in each of the graphs. Data presented here are the mean and standard errors of at least 24 replicates for each cross. Bars with the same letter are not significantly different at *P ≤* 0.05. All of the analyses were done using non-transformed raw aflatoxin data.

The *p2c* transgene was also crossed into 6 elite inbred lines by using pollen from the homozygous and null T4 generation of P2c5 and P2c13 plants to pollinate the elite lines to determine whether the presence of the *p2c* transgene can increase the aflatoxin resistance of the elite lines under field inoculation conditions. Aflatoxin levels in the kernels of crosses between LH210 × P2c5H (homo) or P2c13H were significantly lower compared to those in the kernels from crosses between LH210 × P2c5N (null) or P2c13N with *P ≤* 0.0205 and *P ≤* 0.0039, respectively (**Figure 8A**). The aflatoxin level in the kernels of crosses between PHN46 × P2c5H or P2c13 was significantly lower than that in the control kernels of cross between PHN46 × P2c5N with *P ≤* 0.0019 (**Figure 8B**). The kernels of crosses between PHW79 × P2c5H (homo) or P2c13H also produced significantly less aflatoxin compared to those in the kernels of PHW79 x P2c5N (null) or P2c13N with *P ≤* 0.0097 and *P ≤* 0.0001, respectively (**Figure 8C**). The highest aflatoxin reduction was observed in the cross between PHW79 × P2c13, which is about 83%. In crosses with stiff stalk lines (LH195, LH197, and PHG39), the resulting kernels of LH195, LH197, and PHG39 × P2c5H or P2c13H crosses produced significantly less aflatoxin under field inoculation conditions compared to those in the kernels of LH195, LH197, and PHG39 × P2c5N (null) or P2c13N crosses with *P* values ranging from 0.0001 to 0.0101 (**Figures 8D–F**). The reduction in aflatoxin in the resulting crosses with stiff stalks ranged from 93.7% in PHG39 × P2c13 to 48% in LH197 × P2c5. It is also clear that the crosses with P2c13 have lower aflatoxin than those with P2c5.

## 4 Discussion

The present study examined changes in aflatoxin resistance of T1 and T2 kernels from 11 independent events, and four of them were further self-pollinated to homozygous T3 and T4 generations for further studies. The homozygous transgenic T3 and T4 kernels from P2c5 and P2c13 events showed significant reduction in aflatoxin production under field and laboratory inoculation conditions. When the RNAi-based *p2c* transgene from these two events was transferred into the elite inbred lines, the resulting crosses also exhibited enhanced aflatoxin resistance under field inoculation conditions, demonstrating the observed enhanced aflatoxin resistance in the homozygous transgenic lines was due to the presence of the *p2c* transgene introduced through the HIGS construct. Significant reduction in fungal growth in the inoculated homozygous transgenic maize kernels was also observed (27~40 fold) in P2c5 and P2c13 compared to that in the inoculated non-transgenic P2c5 null and P2c13 null kernels, leading to the observed reduced aflatoxin production in the transgenic kernels since *p2c* does not have any known roles in aflatoxin biosynthesis. Further small RNA sequence analysis revealed that the non-inoculated homozygous leaf and kernel tissues of these two transgenic events contained high levels of *p2c*-specific small RNAs compared to the null and B104 controls. Introducing the *p2c* silencing cassette into elite background through crossing with elite inbred lines also enhanced the aflatoxin resistance in the F1 crosses. These results clearly demonstrated that HIGS construct targeting *p2c* can be used to effectively mitigate *A. flavus* infection and aflatoxin contamination in maize.

The detection of *p2c*-specific small RNAs in the null kernel tissues of T4 P2c5 and P2c13 lines is likely due to the presence of low levels of *A. flavus* infection in the immature kernels since the samples were collected from field-grown plants. This study also demonstrates that the presence of *p2c* transgene did not cause any clear negative impact on average number or weight of kernels per ear, nor any morphological or developmental abnormalities in these HIGS transgenic lines when compared with the wild type (B104) and null.

During the initial evaluation of 11 different transformation events and during in depth evaluation of the progenies from P2c5 and P2c13 events, it was observed that aflatoxin reduction varies greatly from one independent event to another. The transgenic kernels from the P2c13 event always had lower aflatoxin than those from the P2c5 event. One possible reason might be the copy number effect. Initial determination of the number of target *p2c* gene integration using PCR and chi-square analysis indicated that P2c5 might have multiple integrations of the target gene (**Supplementary Table S3**). The droplet digital PCR analysis confirmed that the P2c5 event has 2 copies of *p2c* gene and P2c13 has a single copy of *p2c*. The data from droplet digital PCR pointed to a possible negative dosage (copy number) effect on levels of resistance. To confirm our theory, the small RNA libraries were constructed and sequenced from P2c5 (T0 and T3 leaf and T4 kernel tissues) and P2c13 (T0 leaf and T4 kernel tissues). It was clear that immature kernels from P2c5 produced less small RNA than those from P2c13 (**Table 2**). Although higher level of *p2c* specific small RNA was detected in the leaf tissues of P2c5, it did not seem to have an impact on *A. flavus* infection of the kernel tissue. The consistently lower aflatoxin production observed in homozygous kernels of P2c13 relative to homozygous kernels of P2c5 under field conditions, therefore, could be a combination of significantly higher levels of gene specific sRNAs in the kernel tissue and higher percentage of the 22 nt-long *p2c*-specific sRNAs in P2c13 kernels. The 22 nt small RNA is considered as a more “transitive” silencing signal (Chen et al., 2010b) and has been reported to play a crucial role in gene silencing through amplification of the silencing signal (McHale et al., 2013; Shao et al., 2015; Dalakouras et al., 2018; Dalakouras et al., 2020). Another possible reason for P2c5 always producing more aflatoxin than P2c13 could be due to the differences in locations of the integration of the T-DNA into the genome between the two events (position effect). Increasing the expression levels of these gene specific small RNAs in the kernel tissues through the use of a seed-specific promotor in future studies may further enhance maize aflatoxin resistance.

Evaluation of maize aflatoxin resistance in the field has been difficult. One of the reasons is that the natural infection of maize kernels by *A. flavus* is often sporadic and varies greatly from year to year, which cannot provide a reliable assessment of changes in maize aflatoxin resistance under field conditions. As a result, different artificial inoculation methods have been developed in the past to evaluate maize aflatoxin resistance, such as pin-bar inoculation, side-needle inoculation, and silk channel inoculation (Marsh and Payne, 1984; Campbell and White, 1994; Williams et al., 2013). Different inoculation techniques would influence the route of infection and, potentially, disease severity by altering how the fungus enters the kernel and which tissue it comes into contact with initially (Dolezal et al., 2013; Williams et al., 2013). Although pin-bar inoculation ensures a higher success rate of infection in the field, it should be recognized that this method is invasive since it compromises the kernel’s natural defenses by penetrating the pericarp and aleurone layers, and allowing mycelia to directly grow into the endosperm of many pin-bar wounded kernels (Parsons and Munkvold, 2010; Dolezal et al., 2013). In this study, a less invasive side-needle inoculation method was used to allow infection of immature maize kernels more closely simulating natural conditions. This method has also been widely used by others (Windham and Williams, 1998; Williams et al., 2013).

The average 60-90% reduction in aflatoxin contamination reported in this study was based on three years of field and laboratory inoculation studies to minimize the environmental impact on host resistance and on *A. flavus* virulence. It has been well documented that high temperature, drought, and insect damage promote *A. flavus* infection and aflatoxin production while reducing maize resistance (Marsh and Payne, 1984; Warburton and Williams, 2014). However, the aflatoxin levels in the inoculated transgenic lines as well as in the F1 crosses with the elite inbred lines are significantly lower than their controls, but are still much higher than the maximum level of 20 ppb limit set by FDA for interstate commerce. Part of the reason for these high levels of aflatoxin in the inoculated samples was due to the extremely high concentration of inoculum used in the study (4x10^6^ to 1x10^5^ conidia/mL). We believe the aflatoxin levels in these transgenic lines under natural infection will be much lower since the conidia concentration of *A. flavus* in the air under natural infection conditions is likely to be a million times less than the inoculum used in the present study (Schweer et al., 2016). In addition, only the four kernels surrounding the inoculation sites were collected and analyzed in all of our field studies except the F1 crosses and T3 kernels from P2c7 and P2c17 produced in 2018, in which half of the inoculated ears was ground and subsamples were used. This is another reason for extremely high levels of aflatoxin reported here compared to aflatoxin data from other labs (Mylroie et al., 2016; Windham and Williams, 2016; Womack et al., 2020), which normally grind the whole ear and use subsamples for aflatoxin analysis.

Considering the biological function of *p2c* is to play a part in helping the fungus to breach kernel physical barriers, it was surprising to observe a significant reduction in aflatoxin production in the transgenic maize lines when the kernel physical barrier was breached during needle inoculation, suggesting *p2c* activity is needed by the pathogen to spread from one kernel to another after *A. flavus* enters the maize kernels at the site of inoculation. This speculation is supported by the fact that the inoculated homozygous transgenic kernels of P2c5 and P2c13 have 27 and 40-fold less of *A. flavus* growth compared to that in the inoculated non-transgenic control kernels (**Figure 7**). A recent study by Wu et al. (2022) observed similar results when targeting an endo-polygalacturonase (*SsPG1*) from *Sclerotinia sclerotiorum* through HIGS in transgenic *Brassica napus*, which suppressed *S. sclerotiorum* growth, and appressorium formation.

How gene specific dsRNA travels from the host plant into fungal pathogen is still not well understood. Recent studies found small RNAs including both siRNA and micro RNA (miRNA) may travel between cells in the host plants via plasmodesmata and systemically via phloem (Dunoyer et al., 2010; Pyott and Molnar, 2015). The siRNA was also found to travel into pathogens during infection of host plants and bind to its target gene, leading to the cleavage of its corresponding mRNA into 20-25 nt in size, which results in suppression of pathogen growth and disease development (Großhans and Filipowicz, 2008). Extracellular vesicles have been found to be involved in small RNA trafficking between the host and the pathogen (Cai et al., 2018; Cai et al., 2021). Furthermore, several recent studies demonstrated that exogenously applied dsRNAs can be taken up directly by pathogens and can be used to manage plant fungal diseases (Koch et al., 2016; Wang et al., 2016; Wang and Jin, 2017; Wang et al., 2017; McLoughlin et al., 2018). This spray-induced gene silencing (SIGS) approach can be another effective way to manage aflatoxin contamination in maize if we can better understand how dsRNA is taken up by leaf or silk tissues and transported from maize to *A. flavus*.

## Data availability statement

The original contributions presented in the study are publicly available. This data can be found here: https://www.ncbi.nlm.nih.gov/bioproject/PRJNA945855.

## Author contributions

YR and Z-YC designed the research; YR, OO, QW, SP, and LP performed the research; YR and DH analyzed the data; YR and Z-YC wrote the paper; KW directed the production of the maize transgenic lines; JC and KR provided technical support.
