# Supplementary material for: Targeting the Aspergillus flavus p2c gene through host-induced gene silencing reduces A. flavus infection and aflatoxin contamination in transgenic maize
Source: Front Plant Sci. 2023 May 9;14:1150086. doi: 10.3389/fpls.2023.1150086 (PMC10203651; doi:10.3389/fpls.2023.1150086)
Supplement: Supplementary file 1 [file DataSheet_1.docx]

**Supplemental Figure 1:** Construction scheme of polygalacturonase gene (*p2c*) silencing vector. The pBS-d35S- R4-R3 vector containing a double 35S promoter, followed by an attR4-ccdB-CmR-attR3 cassette amplified from pDEST^TM^ R4-R3 (Invitrogen, Carlsbad, CA) in the pBluescript SK- was constructed in an earlier study (Chen et al., 2010). The DNA regions corresponding to the *p2c* 5′arm and 3′ arm were amplified by PCR with primers containing unique homologous recombination sites cloned into their corresponding entry vectors. They were recombined with pDONR221-PR 10-intron-CmR (Chen et al., 2010) containing a chloramphenicol resistance gene (CmR) selection marker in the middle of the PR10 intron through the LR clonase reaction to assemble the RNAi cassette into the pBluescript vector to produce the pBS-d35S-attB4-5′arm-attB1-PR10 intron-CmR-attB2-3′arm-attB3 vector (named pBS-II-P2c-RNAi). The RNAi cassette was then cloned into the pTF102 vector through ligation to produce the final pTF102-P2c-RNAi vector. This figure was modified from Chen et al. (2010).

**
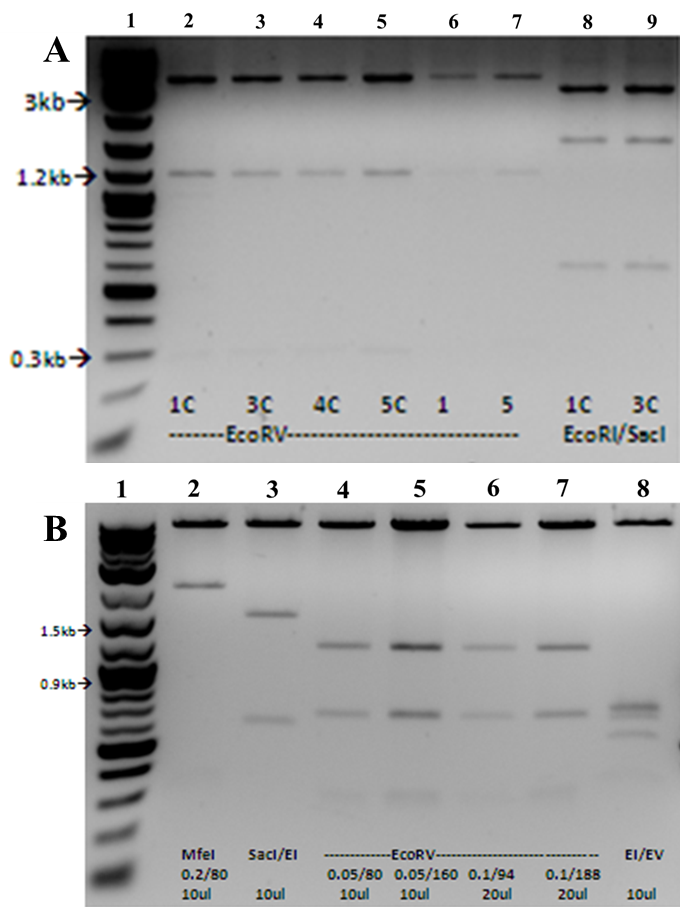
**

**Supplemental Figure 2:** Restriction digestion of the pBS II-P2c-RNAi construct to confirm its correct assembly. Lanes 2-7 were clones digested with *Eco*RV, which resulted in the expected sizes of 262, 1156 and 3589 bp; and lanes 8-9 were clones 1C and 2C digested with *Eco*RI/*Sac*I, which resulted in the expected sizes of 573, 1563, and 2871 bp. B. Restriction digestion of the final pTF102-P2c-RNAi construct with *Mfe*I, which resulted in the expected sizes of 299, 2086 and 8786 bp (lane 2); *Sac*I/*Eco*RI, which resulted in the expected sizes of 573, 1563 and 9035 bp (lane 3); *Eco*RV, which resulted in the expected sizes of 262, 668, 1156 and 9085 bp (land 4-7); and *Eco*RI/*Eco*RV, which resulted in the expected sizes of 262, 479, 668, 677 and 8461 bp (lane 8).


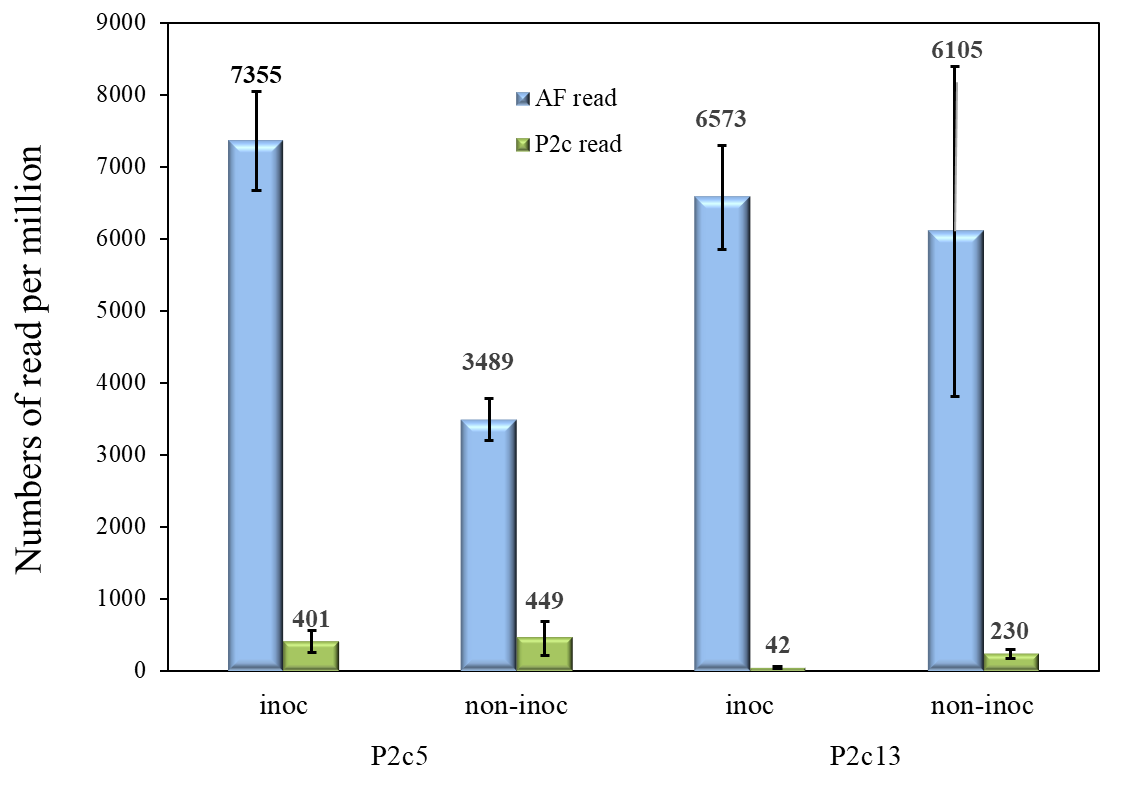


**Supplementary Figure 3:** Analysis of RNAseq data from non-inoculated and inoculated immature maize kernels of T4 generation homozygous P2c5 and P2c13 events collected at 10 days after inoculation with *A. flavus* under field condition. RNA libraries were constructed from total RNAs isolated from P2c5 and P2c13 kernels, sequenced and mapped to *A. flavus* genes as well as to *p2c*. The expression levels (expressed as reads per million) of *A. flavus* specific genes and *p2c* specific genes in the inoculate and non-inoculated P2c5 and P2c13 kernels were presented here.

**Supplemental Table 1**. List of primers used in chapter 3 for constructing the Ti vector for suppressing the *p2c*, zygosity testing and droplet digital PCR

| Primer name | *Oligonucleotide sequence (5’→3’)* |
| --- | --- |
| P2c-F | *GATTACGAGAACGGCAGCC* |
| P2c-R | CAAGAAGCACCAGTAGGAACG |
| RT-P2c-F | CGCTACTGACATCTACATTCTCTG |
| RT-P2c-R | GCACTTGGAGCTAGTCTTACC |
| RT-Zm18S-F | GAGAAACGGCTACCACATCCA |
| RT-Zm18S-R | ACGCGCCCGGTATTGTTAT |
| attB4-P2cF | *GGGGACAACTTTGTATAGAAAAGTT*GATTACGAGAACGGCAGCC |
| attB1-P2cR | *GGGGACTGCTTTTTTGTACAAACTTG*CAAGAAGCACCAGTAGGAACG |
| attB2-P2cF | *GGGGACAGCTTTCTTGTACAAAGTGG*GTTCTCGCACTTGGAGCTAGTC |
| attB3-p2cR | *GGGGACAACTTTGTATAATAAAGTTG*GATTACGAGAACGGCAGCC |
| d35S-F | ATGACGCACAATCCCACTATCCT |
| RNAi-R | GCCATACGGAATTCCGGA |
| PR10-F | CACCTCAGTCATGCCGTTCA |
| RT-P2c-F (Taq) | CGACGCTACTGACATCTACATT |
| RT-P2c-R (Taq) | CGCACTTGGAGCTAGTCTTAC |
| RT-P2c-probe* | FAM-CCTGGCCCG/ZEN/CTGTTTCCATCACT-3IABkFQ |
| RT-Adh1-F (Taq) | CGTCGTTTCCCATCTCTTCCTCC |
| RT-Adh1-R (Taq) | CCACTCCGAGACCCTCAGTC |
| Adh1-probe * | FAM-AATCAGGGC/ZEN/TCATTTTCTCGCTCCTCA-3IABkFQ |
| RT-Bar-F | GGAAGTTGACCG TGCTTGT |
| RT-Bar-R | GATCTACCATGAGCCCAGAAC |
| Bar-probe* | FAM-CGATGTAGT/ZEN/GGTTGACGATGGTGCA-IBFQ |
| RT-Af2-F (its1) | ATC ATT ACC GAG TGT AGG GTT CCT |
| RT-Af2-R (its1) | GCC GAA GCA ACT AAG GTA CAG TAA A |
| Af2-probe* | FAM-CGA GCC CAA /ZEN/CCT CCC ACC CG-3IABkFQ/ |

Italics indicate the homologous recombinant site that attached to the end of the gene specific primer sequences. *: the probes were labeled with FAM (6-fluorescein) or HEX (hexachloro fluorescein) at the 5′end and double quenched with ZEN and Iowa Black FQ (IBFQ).

**Supplemental Table 2**. Variations in the number and average weight of T1 kernels among the 28 transgenic lines from 15 transgenic events of HIGS-p2c in B104 that were produced at Iowa State University.

| Events | Line name | Number of kernels per ear | Kernel weight (g) *  (average) |
| --- | --- | --- | --- |
| P2c3^§^ | P2c3-2 | 180 | 0.1950 |
|  | P2c3-3 | 31 | 0.2289 |
| P2c4^§^ | P2c4-1 | 101 | 0.2306 |
|  | P2c4-2 | 177 | 0.2316 |
|  | P2c4-3 | 203 | 0.1729 |
| P2c5 | P2c5-1 | 246 | 0.1683 |
|  | P2c5-2 | 25 | 0.1918 |
| P2c6 | P2c6-1 | 151 | 0.2324 |
|  | P2c6-2 | 135 | 0.1911 |
| P2c7 | P2c7-1 | 99 | 0.2575 |
|  | P2c7-2 | 126 | 0.1888 |
|  | P2c7-3 | 190 | 0.2642 |
| P2c8 | P2c8-1 | 62 | 0.2515 |
| P2c10 | P2c10-1 | 156 | 0.2250 |
| P2c11 | P2c11-3 | 13 | 0.1765 |
| P2c13 | P2c13-2 | 76 | 0.2433 |
| P2c14 | P2c14-1 | 189 | 0.2180 |
|  | P2c14-2 | 154 | 0.1844 |
|  | P2c14-3 | 215 | 0.1711 |
| P2c17 | P2c17-1 | 40 | 0.2274 |
| P2c18 | P2c18-1 | 42 | 0.2070 |
| P2c21 | P2c21-1 | 26 | 0.2652 |
|  | P2c21-3 | 168 | 0.1797 |
|  | P2c21-4 | 138 | 0.1950 |
| P2c25 | P2c25-1 | 56 | 0.2289 |
|  | P2c25-2 | 16 | 0.2306 |
| P2c29 | P2c29-1 | 52 | 0.2316 |
|  | P2c29-2 | 53 | 0.1729 |

^§^ These two events were negative for the transformation based on PCR.

**Supplemental Table 3**. Number of transgene integration analysis based on genotyping of seedlings developed from self-pollinated T3 generation ears and chi-square analysis

| Event | Number of seedlings | Seedling containing transgene | Seedling without transgene | X^2^ | P | Number of transgene integrations |
| --- | --- | --- | --- | --- | --- | --- |
| P2c5 | 102 |  |  |  |  |  |
| Observed |  | 64 | 38 |  |  |  |
| Expected (1 copy) |  | 76.5 | 25.5 | 8.02 | 0.0046 | ≠1 |
| Expected (2 copies) |  | 95.6 | 6.3 | 169.95 | <0.0001 | ≠2 |
| P2c7 | 51 |  |  |  |  |  |
| Observed |  | 31 | 20 |  |  |  |
| Expected (1 copy) |  | 38.1 | 12.7 | 5.55 | 0.0184 | ≠1 |
| Expected (2 copies) |  | 47.8 | 3.1 | 98.03 | <0.0001 | ≠2 |
| P2c13 | 98 |  |  |  |  |  |
| Observed |  | 77 | 21 |  |  |  |
| Expected (1 copy) |  | 73.5 | 24.5 | 0.66 | 0.4142 | 1 |
| Expected (2 copies) |  | 91.87 | 6.12 | 38.58 | <0.0001 | ≠2 |
| P2c17 | 74 |  |  |  |  |  |
| Observed |  | 59 | 15 |  |  |  |
| Expected (1 copy) |  | 55.5 | 18.5 | 0.88 | 0.3474 | 1 |
| Expected (2 copies) |  | 69.37 | 4.62 | 24.85 | <0.0001 | ≠2 |

Number of transgene integrations was estimated based on probability of calculated chi-square Χ^2^ = ∑ (observed-expected)^2^ / (expected) exceeding the critical value to reject or accept the null hypothesis of being one or two integrations. Segregation of seedlings with transgene and without (null) is expected to be 3:1 (transgene: null) for single integration or 15:1 for two integrations.
